# Depression Reappraisal and Treatment Effect: Will Response Shift Help Improve the Estimation of Treatment Efficacy in Trials for Mood Disorders?

**DOI:** 10.3389/fpsyt.2019.00420

**Published:** 2019-06-27

**Authors:** Samuel Bulteau, Anne Sauvaget, Antoine Vanier, Jean-Marie Vanelle, Emmanuel Poulet, Jérome Brunelin, Véronique Sebille

**Affiliations:** ^1^INSERM-U1246 SPHERE University of Nantes and University of Tours, Nantes, France; ^2^CHU Nantes, Department of Addictology and Psychiatry, Nantes, France; ^3^Laboratory “Movement, Interactions, Performance” (E.A. 4334), Faculty of Sport Sciences, University of Nantes, Nantes, France; ^4^INSERM-U1028, CNRS-UMR5292, Lyon Neuroscience Research Center, ΨR2 Team, University of Lyon, CH Le Vinatier, Lyon, France; ^5^Department of Emergency Psychiatry, Edouard Herriot Hospital, Hospices Civils de Lyon, Lyon, France; ^6^CHU Nantes, Department of Methodology and Biostatistics, Nantes, France

**Keywords:** depression, reappraisal, patient-reported outcomes, response shift, efficacy

Major depressive disorder (MDD) has the highest burden of all mental health disorders. It affects more than 300 million people worldwide and is a leading cause of disability. Beyond the classical focus on increased negative emotions, self-referential thoughts may play a crucial role in depression (1, 2). Indeed, individuals with MDD tend to interpret their experiences with a negative cognitive bias toward the self, have higher levels of self-consciousness in relation to depression severity, and have enhanced memory for one’s own negative personality traits (3, 4). Moreover, the neural architecture underpinning the tendency to overgeneralize self-blaming in MDD has recently been elucidated (5), and this aberrant self-referential processing may be an important factor in the mechanism of action of antidepressants (while shifting the focus from the self to the external environment and enhancing positive self-referential processing) (6).

Changes in self-perception and changes in the disease or treatment effect reappraisal over time may have an impact on self-assessment of MDD.

The most widely used self-report scale to measure depression and assess treatment efficacy is the BDI (Beck Depression Inventory). Its psychometric properties allow accurate discrimination between depressed and non-depressed populations and a good estimation of depression severity (7). Longitudinal data are often collected to assess changes in the BDI scores, and it is usually assumed that the scale measures MDD in the same way over time (longitudinal measurement invariance. However, the assumption of longitudinal measurement invariance may sometimes be compromised and impact conclusions (8).

Our belief is that treatment has a direct effect on outcomes but also an indirect impact *via* treatment-induced changes in appraisal in the interpretation of the items of the scale, or even the concept of being measured (depression). For example, Fokkema and colleagues (9) suggested, using confirmatory factor analysis on the sample of the National Institute Mental Health Treatment of Depression Collaborative Research Program (*n* = 155), that participants got better at assessing their levels of depressive symptoms and have become also more aware of them after treatment.

This is supported by the recent demonstration, on the basis of influential randomized controlled trials in the field such as sequenced treatment alternatives to relieve depression (STAR*D) or Netherlands study of depression and anxiety (NESDA) studies (*n* = 3,509). These studies showed that the instruments analyzed in this report [Hamilton Rating Scale for Depression, Inventory of Depression Symptomatology (IDS)-Clinician rated, Quick Inventory of Depression Symptomatology, and IDS-Self Rated] did not assess a single underlying construct and did not measure the same (set of) construct(s) in the same way across time. This could be indicative of reconceptualization explained by changes in depression’s perception and meaning before and after treatment (10). Hence, we support the opinion that treatment effect (considered as “true change”) may be underestimated (or overestimated) due to a reappraisal of the disease and treatment impact.

This raises an important question regarding reliable and unbiased depression assessment since the influence of MDD and treatment on self-evaluation changes over time may lead to draw erroneous conclusions regarding treatment efficacy. If we study treatment efficacy using a method assuming no appraisal modifications over time, we might not optimally assess outcomes.

Response shift (RS) reflects the patients’ changes in the meaning of their self-evaluation [beyond the evaluation of the construct the patient-reported outcomes (PRO) aims to measure]. It allows disentangling true change and modification of self and disease perception over treatment course, and offers better understanding of those changes in PRO assessment (11–13).

The study of RS can help to detect changes in patient’s reappraisal of his/her symptomatology and to identify patient’s subgroups that may be vulnerable to poor unchanged perceived improvement despite objective improvement observed by the clinician. RS has already been widely studied in quality of life research in several clinical studies in oncology or neurology, but to date there are very few empirical studies in MDD (14, 15).

RS is currently operationalized in three different forms:

- *Recalibration:* change in the respondent’s internal standards of measurement (e.g., change in the interpretation of the response categories of items of the scale). For instance, after 6 months of treatment, “feeling guilty most of the time” does not correspond to the same level of guilt than at diagnosis. This can also occur when patients get better at assessing their level of depressive symptoms. For example, three recent studies (10, 16, 17) using Structural Equation Modeling (SEM), which depicts relations among observed variables and latent variables (construct), demonstrated the impact of recalibration on self-rated depression levels. In a clinical trial comparing antidepressants and psychotherapy (*n* = 155), recalibration RS caused overestimation of the depressive symptomatology after treatment (i.e., underestimation of true change) as compared to pre-treatment assessment (10). Furthermore, the authors reported that patients who received a psychotherapy experienced RS to a greater extent than patients who received medication. Elhai and colleagues found clear recalibration signs suggesting underestimation of true change in a sample of 1,025 depressed adult patients (16). Wu and colleagues evidenced recalibration on BDI scores (primarily in negative attitude factor) in a sample of 320 adolescents but in an opposite direction (BDI-II factor scores underestimated in that study) (17).
- *Reprioritization:* change in the importance of different factors of depression, more precisely changes in relative importance of component domains (e.g., performance impairment becoming more important than sad mood) in constituting the target construct (depression). Reprioritization might occur when a subject experiences a particular life or health event that changes his/her priorities or the attention drawn to several particular symptoms rather than others. The results reported in the literature can be heterogeneous. While Fokkema and colleagues (9) and Elhai and colleagues (16) showed reprioritization, Wu and colleagues (17) did not. This discrepancy may be explained by the differing population samples with a more prominent perception of treatment effect as a relevant health event in clinical sample of depressed adults (in the two former studies) than in non-clinical student sample (in the latter).
- *Reconceptualization:* change in the patient’s definition of what is being measured (respondents redefine the concept of depression and which components are contained in the construct). For example, after depression remission, a patient considers the items related to previously unrecognized somatic factors as an important part of depression. Literature about reconceptualization in MDD is rare so that it is difficult to assume how it can influence true change in MDD trials.

Two main approaches have been proposed to detect and estimate RS:

1. Methods based on specific study design including either specific measurement tools such as the Quality of Life Appraisal Profile (QOLAP) or retrospective ratings of baseline assessment (thentest), and qualitative interviews (18–20),
2. Statistical methods, including SEM, Item Response Theory (IRT), and Rasch Measurement Theory models (RMT), that can be used to analyze data and do not require a specific design (12, 21).

A recent scoping review (22) has shown that the most commonly used approaches were SEM and the thentest. SEM enables detecting all forms of RS (recalibration, reprioritization, reconceptualization). The thentest can detect recalibration RS thanks to a retrospective evaluation of a previous assessment, but is prone to recall bias. Other methods have been proposed such as IRT and RMT for recalibration and reprioritization RS detection at the item-level, or relative importance analysis based on logistic regression for reprioritization RS detection at the domain level (23). SEM operationalizes RS as changes in SEM parameters over time as follows: change in intercepts (uniform recalibration), residual variances (non-uniform recalibration), factor loadings (reprioritization), or factor structure (reconceptualization).

We wish to underline the value of RS analysis in former or future clinical data in order to estimate and account for under- or over-estimation of true change as well as to identify clusters of patients more prone to have a self-reported under- or over-estimation of their depressive symptomatology. Randomized clinical trial (RCT) methodology could be improved by integrating more baseline descriptive data to get more insight into patients’ experience over the treatment course using RS analyses. According to the model of Sprangers and Schwartz (11), RS usually depends on a catalyst (e.g., health event) on several individual’s characteristics, such as existing beliefs, expectations, goals, and psychological mechanisms (e.g., behavioral, cognitive, and affective coping processes). For example, paying attention to personal goals, through personal goal attainment scaling (GAS), may be of importance in RCT for MDD since goal definition and perception is relevant for MDD prognosis and is likely to be linked with RS (24, 25). Hence, it could be of value to collect variables related to underlying personality, potential malignant self-regard, insight, illness perception, and beliefs related to medical history (chronicity, resistance, etc.) to better interpret self-reported change and RS occurrence, if appropriate. One can assume that some patients with some types of personality are more prone to change their evaluation over time, while other may be more “persistent” in their evaluation, and that baseline coping style has a great influence on reappraisal process. After treatment, an analysis of lived experience perspective with qualitative approaches (e.g., semi-structured interviews) may help to find narrative descriptions reflecting and explaining changes in values and internal standard (26).

Moreover, studying RS effect (especially reprioritization or reconceptualization) may help in focusing on residual symptoms whose relative importance depends on the patient’s personal objectives and priorities and may change over time. Detection of reprioritization or reconceptualization RS may indeed highlight changes in the importance of some clinically relevant residual symptoms that may otherwise go undetected and increase risk of relapse. Eventually, RS may depend on the factors that are more sensitive to it (e.g., negative attitude, performance impairment, or self-blame) and on the type of therapeutic intervention (cognitive behavioral approach, medication, brain stimulation targeting specific networks). Investigating RS effect in clinical studies may help in identifying subgroups of patients in need for more support or more prone to benefit from some synergistic approaches (e.g., cognitive remediation and antidepressants) and thus contribute to precision psychiatry.

## Author Contributions

SB and VS analyzed literature and wrote the manuscript. AV and VS afforded methodological expertise. EP, JB, JMV and AS gave their viewpoint as clinicians and researchers and contributed to manuscript improvement.

## Conflict of Interest Statement

The authors declare that the research was conducted in the absence of any commercial or financial relationships that could be construed as a potential conflict of interest.
